# Modification of Eye–Head Coordination With High Frequency Random Noise Stimulation

**DOI:** 10.3389/fnhum.2020.592021

**Published:** 2020-11-20

**Authors:** Yusuke Maeda, Makoto Suzuki, Naoki Iso, Takuhiro Okabe, Kilchoon Cho, Yin-Jung Wang

**Affiliations:** ^1^Department of Physical Therapy, School of Health Sciences at Odawara, International University of Health and Welfare, Kanagawa, Japan; ^2^Faculty of Health Sciences, Tokyo Kasei University, Saitama, Japan; ^3^Day-care Center, Yamagata, Japan

**Keywords:** vestibulo-ocular reflex, eye–head coordination, fixation, lag time, high frequency noisy vestibular stimulation

## Abstract

The vestibulo-ocular reflex (VOR) plays an important role in controlling the gaze at a visual target. Although patients with vestibular hypofunction aim to improve their VOR function, some retain dysfunction for a long time. Previous studies have explored the effects of direct current stimulation on vestibular function; however, the effects of random noise stimulation on eye–head coordination have not previously been tested. Therefore, we aimed to clarify the effects of high frequency noisy vestibular stimulation (HF-nVS) on eye–head coordination related to VOR function. Thirteen healthy young adult participants with no serious disease took part in our study. The current amplitude and density used were 0.4 mA and 0.2 mA/cm^2^, respectively, with a random noise frequency of 100–640 Hz. The electrodes were located on both mastoid processes. The stimulus duration and fade in/out duration were 600 and 10 s, respectively. Subjects oscillated their head horizontally, gazing at the fixation point, at 1 Hz (0.5 cycles/s) for 30 repetitions. The coordination of eye–head movements was measured by eye-tracking and a motion capture system. Peak-to-peak angles for eye and head movement and deviation of the visual line from the fixation target revealed no significant differences between HF-nVS and sham. The lag time between the eye and head movement with HF-nVS post-stimulation was significantly shorter than that of the sham. We found that HF-nVS can reduce the lag time between eye and head movement and improve coordination, contributing to a clear retinal image. This technique could be applied as a form of VOR training for patients with vestibular hypofunction.

## Introduction

When we look at an object in the world around us, eye–head coordination is vital to accurately identifying that object. The vestibulo-ocular reflex (VOR) functions to correct eye movements during head movement and leads to the stable and sharp foveal vision of the object (Chin, 2018). The vestibula, including three semicircular canals and otolith organs (utricle and saccule), can perceive angular velocity and the acceleration of the head (Yang et al., 2015). The VOR receives positional input from the vestibular afferents (Ischebeck et al., 2017, 2018) and sends signals to the eye muscles and cerebellum, which send feedback signals to modulate or fine-tune the VOR (Wallace and Lifshitz, 2016). Thus, the position of the eyes affects the movement of the head.

It is well known that the vestibular nerves associated with both semicircular canals and otolith organs can be electrically stimulated (Fitzpatrick and Day, 2004; Uchino and Kushiro, 2011; Yang et al., 2015). Several previous studies applied a small direct current to the right and left mastoid processes behind the ear, and the firing rate of all vestibular afferents could be modified by the current (Fitzpatrick and Day, 2004; Uchino and Kushiro, 2011; Yang et al., 2015). Recently, Forbes et al. (2020) demonstrated that vestibular afferents were sensitive to alternating currents ranging randomly between low and high frequencies (0–300 Hz) known as Noisy Vestibular Stimulation, and the neck motor neurons were activated by vestibular stimulation. In addition, a review by Forbes et al. (2014) suggested that noisy vestibular stimulation with low to high frequencies and low amplitude could modify postural control. Moreover, some previous studies have applied low frequency with low amplitude (0–30 Hz and 0.3–0.5 mA, respectively) (Fujimoto et al., 2016; Wuehr et al., 2016) or low to high frequency with low amplitude noise vestibular stimulations (0.1–640 Hz and 0.4–1.0 mA, respectively) (Inukai et al., 2018) over the mastoid process. These studies suggested that noisy vestibular stimulation with low to high frequency with low amplitude improved walking performance (Wuehr et al., 2016) and standing balance (Fujimoto et al., 2016; Inukai et al., 2018). Meanwhile, to stimulate cortical neurons, a high frequency noise stimulation (100–640 Hz) can increase cortical excitation (Terney et al., 2008; Penton et al., 2018; Pavan et al., 2019). Specifically, high frequency random noisy stimulation between 100 and 640 Hz increased cortical neuron excitability, which lasted up to 60 min even after stimulation was stopped (Terney et al., 2008; Inukai et al., 2016). Others have demonstrated that high frequency noisy stimulation improves behavioral performance in visual detection and discrimination (Romanska et al., 2015; Campana et al., 2016; van der Groen and Wenderoth, 2016), perceptual learning (Fertonani et al., 2011; Camilleri et al., 2016; Moret et al., 2018), and arithmetic skills (Snowball et al., 2013; Pasqualotto, 2016; Popescu et al., 2016). In particular, the vestibular afferents were activated in response to high frequency noisy stimulation (Forbes et al., 2020). These results implied that the noise vestibular stimulation at high frequency might affect human behavior, mainly influenced by the vestibula.

Several studies (Uchino and Kushiro, 2011; Yang et al., 2015) and a review (Fitzpatrick and Day, 2004) have reported a direct current or step waveform that also alternates the direction of the current between step pulses. Although some studies have used noisy vestibular stimulation, they only assessed the activation of the vestibular afferents and neck motor neurons (Forbes et al., 2020), walking performance (Wuehr et al., 2016), and standing balance (Fujimoto et al., 2016; Inukai et al., 2018). The voluntary performance of eye–head coordination during high frequency and low amplitude noisy vestibular stimulation was not investigated. Therefore, although high frequency noisy vestibular stimulation (HF-nVS) can influence neural activity, it remains unclear whether eye–head coordination performance is affected by HF-nVS in the context of the VOR function. If, in addition to the knowledge provided by Forbes et al. (2020), the relationship between HF-nVS and eye–head coordination performance could be clarified, then we might understand better the nVS-induced modification processes for eye–head coordination and performance that occur in VOR functional modification. We, therefore, designed a paradigm involving eye–head coordination during HF-nVS. We predicted that if HF-nVS affects the VOR function, then HF-nVS should decrease the deviation from fixation targets and the time lag between the eye and head motions. We, therefore, investigated eye–head coordination during HF-nVS. Exploring how HF-nVS affects eye–head coordination tasks may have interesting implications for VOR training potential in behavioral science and neuroscience.

## Materials and Methods

### Participants

Our target sample size was based on a 90% statistical power to detect changes in eye–head coordination with a 0.90 effect size and a two-sided α-level of 0.05. Inputting these parameters into the Hulley matrix (Hulley et al., 1988) yielded a sample size of 12. We recruited 13 healthy, neurologically intact subjects [two men and 11 women aged 21–48 years, mean ± standard deviation (SD): 33.4 ± 11.2 years] for the eye and head movement measurements. Screening for medication use and medical history was performed through an interview. Here, participants were also informed about the research, such as its purpose; procedures; duration of the experiment; potential risks, adverse effects, or discomfort that may occur; and the right to decline to participate in the study. None of the subjects took medications or had any psychiatric or neurological diseases. Our experimental procedures were approved by the Research Ethics Committee of the Tokyo Kasei University and performed following the principles of the Declaration of Helsinki. All subjects provided written informed consent prior to participation.

### Recording of Reflexive Eye Movements

**FIGURE 1:**
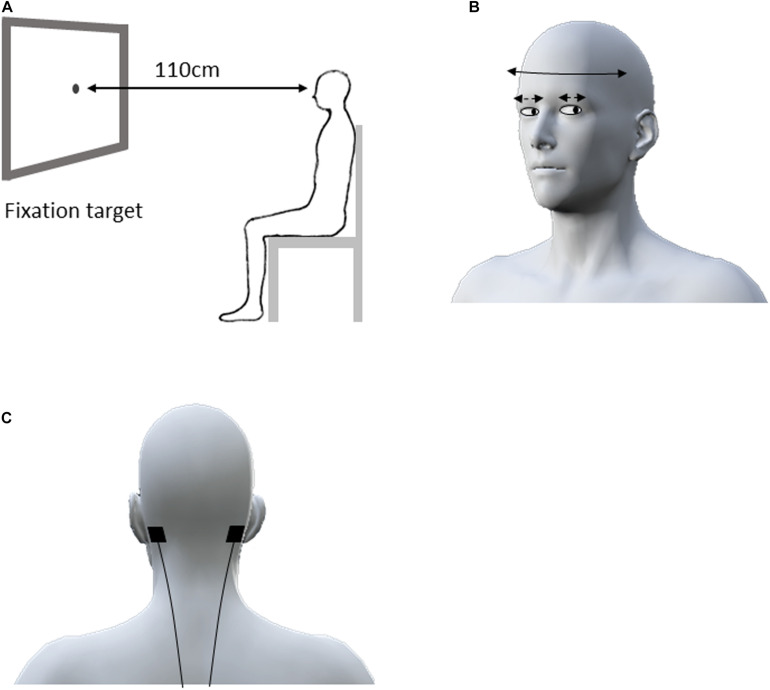
Experimental design for the eye–head coordination task. The subject sat in front of a 0.5 cm fixation target on the computer screen located 110 cm from the face at eye level **(A)**. The subjects were asked to swing their head right and left alternately as far as possible for 30 s in accordance with the rhythm of a metronome at 1 Hz while staring at the fixation target **(B)**. During the eye–head coordination task, the electrodes for active and sham HF-nVS were positioned at either mastoid process **(C)**. HF-nVS, high frequency noise vestibular stimulation.

Each subject sat comfortably in front of a 0.5 cm diameter fixation target on the computer screen located 110 cm from the face at eye level (Figure 1A). Horizontal compensatory eye movement angles in response to sinusoidal horizontal head rotations were measured by an infrared camera (TalkEye Lite, Takei Scientific Instruments Co., Ltd., Tokyo, Japan) from the right eye during eye–head coordination tasks. The head rotation angle was recorded using the VICON motion capture system (Vicon Motion Systems, Ltd., Oxford, United Kingdom). Three infrared reflective markers (14 mm in diameter) were placed on the subject’s forehead. The sampling frequency was 30 Hz for eye tracking and 100 Hz for motion capture, respectively.

In previous studies, the participant’s head was passively turned approximately 5–15° at 0.1–33 s for VOR assessment (Halmagyi et al., 2017; Ischebeck et al., 2017, 2018). However, in daily living, when people actively turn their heads faster and wider and gaze at the fixation target, their eye movement can compensate for the head turn to maintain their gaze on the target (Halmagyi et al., 2017). Therefore, in the eye–head coordination tasks of our study, each participant actively, widely, and horizontally performed head oscillations. Head oscillations occurred, with a gazing fixation point, at 1 Hz (0.5 cycles/s) for 30 repetitions. Specifically, to assess active eye–head coordination performance, we did not passively turn the participant’s head, but rather, asked the participant to horizontally swing his/her head from right to left, as far as possible, for 30 s in accordance with the rhythm of a metronome, while staring at the fixation target (Figure 1B).

### HF-nVS

Stimulation was delivered via a battery-driven electrical stimulator (DC-Stimulator Plus, Eldith, NeuroConn GmbH, Ilmenau, Germany) through 2.0 cm^2^ conductive rubber electrodes with paste. For both active and sham HF-nVS, the electrodes were positioned according to the mastoid process (Fitzpatrick and Day, 2004; Uchino and Kushiro, 2011; Yang et al., 2015; Inukai et al., 2018) (Figure 1C). As previously described (Terney et al., 2008; Inukai et al., 2018; Brevet-Aeby et al., 2019; Donde et al., 2019; Moret et al., 2019; Forbes et al., 2020), noisy stimulation was administered using a device with a current density of 0.06–0.5 mA/cm^2^. We then delivered electrical currents of 0.4 mA to 2.0 cm^2^ via small electrodes to achieve a current density of 0.2 mA/cm^2^ with alternating currents ranging randomly between 100 and 640 Hz (each frequency has equal power as white noise). For active HF-nVS, stimulus duration and fade in/out duration was 600 and 10 s. A previous study (Terney et al., 2008) applied an electrical current to a participant’s skin for a short time, because the participant tended to perceive sensations, such as tingling, at the beginning of stimulation. Thus, in our study, for sham HF-nVS, the short-time stimulus and fade in/out durations were 60 and 10 s, respectively. After 60 s of stimulation, the stimulator was turned off, but electrodes were held at the mastoid process.

### Experimental Procedure

**FIGURE 2:**
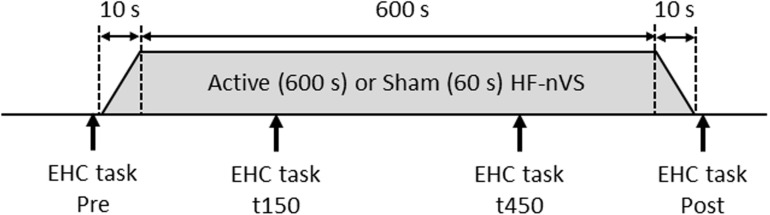
Time course of the experimental procedure. For active HF-nVS, stimulus duration and fade in/out duration were 600 and 10 s. For sham HF-nVS, stimulus duration and fade in/out duration were 60 and 10 s. Each eye–head coordination task was conducted four times. EHC, eye–head coordination; HF-nVS, high frequency random noise stimulation; Pre, before stimulation beginning; t150, 150 s after the stimulation onset; t450, 450 s after the stimulation onset; Post, after the stimulation endpoint.

The time path of the experimental procedure is schematically shown in Figure 2. The repeated-measurement design consisted of a cross-over, in which the two stimulus conditions for active and sham HF-nVSs were randomly performed with a break of at least 1 day in between. Participants experienced both active and sham HF-nVS conditions, and the starting condition was randomly assigned. The eye–head coordination tasks were conducted before (Pre) stimulation, 150 s (t150) and 450 s (t450) after the onset of stimulation (i.e., 160 and 460 s including fade-in duration), and after (Post) the stimulation endpoint (i.e., 620 s after the stimulation beginning including fade in/out duration).

### Data Analysis

All data were visually inspected and removed if contaminated by excessive noise, such as eye blinks. The blank cells produced by removing eye blinks were then linearly interpolated across the blank cells. Eye and head angle changes were then normalized to the difference from the initial angle of each participant, to eliminate inter-individual differences in initial eye and head positions. Next, data were up sampled to 300 Hz by cubic spline function to solve differences in sampling rates between the eye and head recordings.

To measure the deviation of the visual line from the fixation target, head angles were subtracted from eye angles. The absolute values of the visual line deviation were then calculated, resulting in a single number for each period (i.e., data sampled at 300 Hz for 30 s in Pre, t150, t450, and Post). The mean of the absolute values of visual line deviation in each Pre, t150, t450, or Post for each participant was used. Next, each participant’s mean value in Pre was subtracted from the mean values in each t150, t450, or Post to provide normalized visual line deviation as an amount of change at t150, t450, and Post from Pre. To carefully assess intra- and inter-individual changes, differences in the normalized visual line deviations between active and sham HF-nVSs in t150, t450, and Post were analyzed by the permutated Brunner–Munzel test. The permutated Brunner–Munzel test, based on asymptotic permutational distribution, can compare small sample data, leading to a standard normal distribution and accurate *p-value* (Fagerland et al., 2011).

In addition, each subject’s up-sampled data at 300 Hz of eye and head movements were normalized by linear transformation, and the data were expressed as Z scores (Aglioti et al., 2008) because peak-to-peak angles of eye and head movements were different between subjects. To quantify the time lag of compensatory eye movement in response to head movement, the time lag at minimum *r* value between up-sampled Z scores of eye and head movements was identified by cross-correlation function in each Pre, t150, t450, or Post because eye and head angles were expected to be in the opposite phase. After identifying time lag at minimum *r* value by cross-correlation function, the absolute values of the time lag were calculated. Each participant’s time lag in Pre was then subtracted from the time lags in each t150, t450, or Post to provide the normalized time lag. Differences in the time lags between active and sham HF-nVSs in t150, t450, and Post were analyzed by the permutated Brunner–Munzel test. We defined statistical significance as *p* < 0.05. All statistical analyses were performed with R 3.5.2 software (R Foundation for Statistical Computing, Vienna, Austria).

## Results

**FIGURE 3:**
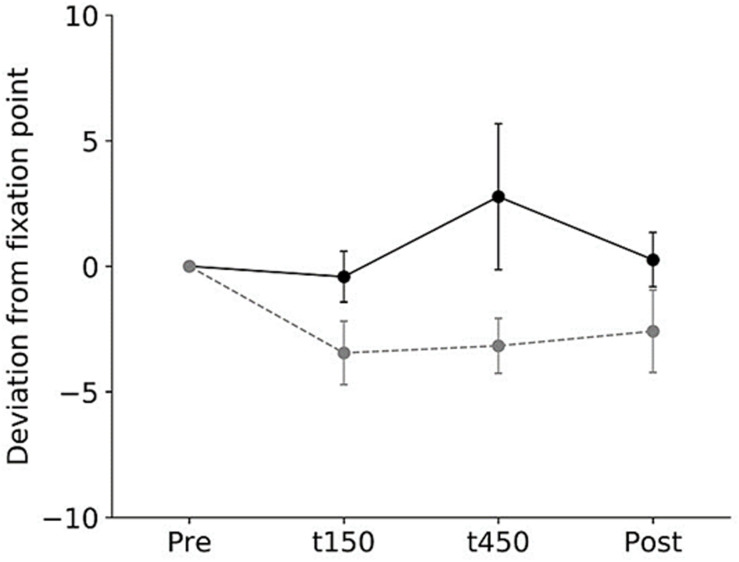
Normalized visual line deviations during active and sham HF-nVSs. Black line and symbols denote active HF-nVS, and gray line and symbols denote sham HF-nVS. The symbols and bars denote the mean and standard error of the mean. The normalized visual line deviations for t150, t450, and Post were not significantly different between active and sham HF-nVS (permuted Brunner–Munzel test, t150, *p* = 0.977; t450, *p* = 0.977; Post, *p* = 0.935). HF-nVS, high frequency noisy vestibular stimulation; Pre, before stimulation onset; t150, 150 s after stimulation onset; t450, 450 s after the stimulation onset; Post, after the stimulation endpoint.

**FIGURE 4:**
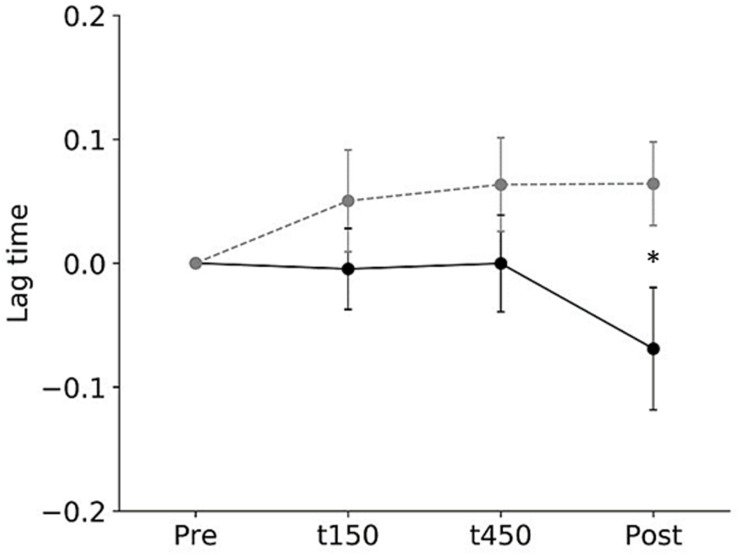
The normalized root-mean-square values of time lags during active and sham HF-nVSs. Black line and symbols denote active HF-nVS, and gray line and symbols denote sham HF-nVS. The symbols and bars denote mean and standard error of the mean. The normalized root-mean-square values of time lags were significantly smaller for active HF-nVS than for sham HF-nVS at Post (permuted Brunner–Munzel test, t150, *p* = 0.204; t450, *p* = 0.222; Post, *p* = 0.030). HF-nVS, high frequency noisy vestibular stimulation; Pre, before stimulation onset; t150, 150 s after stimulation onset; t450, 450 s after the stimulation onset; Post, after the stimulation endpoint.

All subjects completed all experimental conditions. No adverse HF-nVS-related effects occurred during the experiments. Table 1 shows the peak-to-peak angles of the eye and head oscillations from subjects exposed to active or sham HF-nVS. Although both eye and head movements were sinusoidally and horizontally changed, peak-to-peak angles of eye movements were slightly smaller than those of the head. The absolute degree of the deviation of the visual line from the fixation target is shown in Table 2. Mean visual line deviations in the Pre, t150, t450, and Post were approximately 18.1°–22.1°. The visual line deviations normalized by subtraction of the Pre data from t150, t450, and Post were not significantly different between active and sham HF-nVSs (permuted Brunner–Munzel test, t150, *p* = 0.977; t450, *p* = 0.977; Post, *p* = 0.935; Figure 3). Table 3 depicts the time lags and *r* values derived from the cross-correlation function. The *r* values of time lags were consistently negative and high across all experimental groups. The mean time lags in the Pre, t150, t450, and Post were approximately −0.88 to 0.52 s. The time lags normalized by subtraction of the Pre data from t150, t450, and Post were 0.050 ± 0.041, 0.063 ± 0.038, and 0.064 ± 0.034 s for sham HF-nVS, respectively, and were −0.005 ± 0.033, −0.0002 ± 0.039, and −0.069 ± 0.049 s for active HF-nVS, respectively. The permutated Brunner–Munzel test showed that there was a significantly smaller time lag for active HF-tRNS compared to sham HF-tRNS at Post (permuted Brunner–Munzel test, t150, *p* = 0.204; t450, *p* = 0.222; Post, *p* = 0.030; Figure 4).

**TABLE 1:** Peak-to-peak angles for eye and head movements.

	**Conditions**							
	**Sham HF-nVS**				**Active HF-nVS**			
**Peak-to-peak angle (degree)**	**Pre**	**t150**	**t450**	**Post**	**Pre**	**t150**	**t450**	**Post**
Eye Head	49.7 ± 1.1 39.9 ± 0.9	45.1 ± 1.0 35.7 ± 0.8	47.4 ± 0.9 37.9 ± 0.8	48.7 ± 1.0 38.7 ± 0.9	48.6 ± 0.9 39.0 ± 0.8	47.2 ± 0.9 38.3 ± 0.8	47.7 ± 1.0 39.0 ± 0.9	47.7 ± 1.0 38.6 ± 0.8

*Values are mean ± standard error of the mean.HF-nVS, high frequency noisy vestibular stimulation; Pre, before stimulation onset; t150, 150 s after stimulation onset; t450, 450 s after the stimulation onset; Post, after the stimulation endpoint.*

**TABLE 2:** The absolute degree of deviation of the visual line from the fixation target.

**HF-nVS**	**Pre**	**t150**	**t450**	**Post**
Sham Active	21.3 ± 0.06 19.3 ± 0.05	17.9 ± 0.04 18.9 ± 0.05	18.1 ± 0.05 22.1 ± 0.44	18.7 ± 0.05 19.6 ± 0.05

*Values are mean ± standard error of the mean.HF-nVS, high frequency noisy vestibular stimulation; Pre, before stimulation onset; t150, 150 s after stimulation onset; t450, 450 s after the stimulation onset; Post, after the stimulation endpoint.*

**TABLE 3:** Time lags and r values derived from the correlation function.

**HF-nVS**	**Pre**		**t150**		**t450**		**Post**	
	**Time lag (s)**	***r* value**	**Time lag (s)**	***r* value**	**Time lag (s)**	***r* value**	**Time lag (s)**	***r* value**
Sham Active	0.45 ± 0.02 0.51 ± 0.03	−0.83 ± 0.05 −0.88 ± 0.05	0.50 ± 0.04 0.50 ± 0.02	−0.79 ± 0.05 −0.86 ± 0.05	0.52 ± 0.04 0.51 ± 0.04	−0.87 ± 0.04 −0.78 ± 0.08	0.52 ± 0.03 0.44 ± 0.04	−0.85 ± 0.05 −0.85 ± 0.06

*Values are mean ± standard error of the mean.HF-nVS, high frequency noisy vestibular stimulation; Pre, before stimulation onset; t150, 150 s after stimulation onset; t450, 450 s after the stimulation onset.*

## Discussion

To test the hypothesis that HF-nVS should decrease the deviation from the fixation target and the time lag between the eye and head motions, we measured changes in eye–head coordination related to the VOR function. Our results show that the normalized time lag of compensatory eye movement in response to head movement was smaller after active HF-nVS than after sham HF-nVS. The visual line deviation was not different between active and sham HF-nVSs. The normalized time lag between eye and head movements was significantly smaller after active HF-nVS and maintained a stable visual line deviation. This implies that HF-nVS affects eye–head coordination, reflecting the time lag between eye and head movements. This is the first systematic study to show that HF-nVS changes eye–head coordination.

Corrective eye movement in response to head movements is essential for stabilizing foveal vision. The vestibular receptors of the inner ear are known to accurately detect movements of the head (Chin, 2018). The VOR receives input from the vestibular receptors, responding to movements of the head (Ischebeck et al., 2018). Electrical stimulation is a well-known procedure used to stimulate the vestibular system (Fitzpatrick and Day, 2004; Kim and Curthoys, 2004; Uchino and Kushiro, 2011; Yang et al., 2015; Fujimoto et al., 2016; Wuehr et al., 2016; Inukai et al., 2018). A small electrical direct current or step waveform applied to the mastoid process can modulate vestibular nerve activity (Fitzpatrick and Day, 2004; Uchino and Kushiro, 2011; Yang et al., 2015; Chin, 2018; Mackenzie and Reynolds, 2018). Recently, high frequency noisy stimulation has been found to apply to cortical (Terney et al., 2008) and vestibular neurons (Forbes et al., 2014, 2020; Inukai et al., 2018). Moreover, applying a noise small current with 0.05–20 Hz (the amplitude and density values were 10 mA and 0.6 mA/cm^2^, respectively) (Mackenzie and Reynolds, 2018), 0–30 Hz (amplitude, 0.3–0.5 mA) (Fujimoto et al., 2016; Wuehr et al., 2016), and 0.1–640 Hz (the amplitude and density values were 0.4–1.0 mA and 0.2–0.5 mA/cm^2^), respectively (Inukai et al., 2018), to the mastoid area alters ocular torsion response (Mackenzie and Reynolds, 2018), body sway response (Fujimoto et al., 2016; Inukai et al., 2018), and walking performance (Wuehr et al., 2016) related to the vestibular system. We observed that the time lag between eye and head movements was reduced after active HF-nVS over the mastoid process. This is a novel observation from our study.

In our study, one potential effect of a decreased time lag between eye and head movements by HF-nVS may be the stochastic resonance phenomenon. Stochastic resonance is a phenomenon in which the response of a system to an input signal benefits from the presence of noise (Gammaitoni et al., 1989; Collins et al., 1995; Gammaitoni, 1995; Gluckman et al., 1996; Moss et al., 2004; McDonnell and Abbott, 2009; Nobusako et al., 2018). Previous studies have noted that random noise stimulation could enhance the detection of weak stimuli or enhance the sensitivity of neurons to a weak stimulus related to the stochastic resonance phenomenon (Moss et al., 2004; Pavan et al., 2019). Because the effect of stochastic resonance is due to the improvement of signal detection in the presence of noise, stochastic resonance can provide noise benefits to some sensory and motor systems (Moss et al., 2004; McDonnell and Abbott, 2009; Nobusako et al., 2018). The effects of HF-nVS used in our study can be explained within the stochastic resonance framework, that is, the neural noise induced by HF-nVS could increase VOR function and improve eye–head coordination. However, further physiological and behavioral studies are required to understand the effects of HF-nVS on the VOR function.

The small size of the electrodes results in more focused spatial stimulation of the mastoid areas, which may be related to the current densities. Although we used small electrodes and low current amplitude, the current density was not low (0.2 mA/cm^2^) compared to previous studies (0.06–0.5 mA/cm^2^) (Terney et al., 2008; Inukai et al., 2016, 2018; Brevet-Aeby et al., 2019; Donde et al., 2019; Moret et al., 2019; Forbes et al., 2020). In the present study, small electrodes with the optimal current density (0.2 mA/cm^2^) for activating the VOR function were used. In the stochastic resonance phenomenon, a system is characterized by the output signal-to-noise ratio, which is defined as the ratio of the strength of the signal peak to the background noise at the input signal frequency (Collins et al., 1995; McDonnell and Abbott, 2009). Therefore, higher or lower current amplitude leads to a worse response of the system, including the low signal-to-noise ratio and disturbance of signal detection, whereas optimal current amplitude leads to an improved response of the system. In our study, HF-nVS could lead to eye–head coordination change including the VOR function related to optimal weak noise (i.e., optimal stimulus density in accordance with low current amplitude for small electrodes) in the stochastic resonance phenomenon (Moss et al., 2004). There is not, however, sufficient evidence to suggest that the current used in our study can reach the vestibular system. Although we expect that HF-nVS stimulates the vestibular system based on previous studies’ stimulation intensities (Terney et al., 2008; Inukai et al., 2016, 2018; Brevet-Aeby et al., 2019; Donde et al., 2019; Moret et al., 2019; Forbes et al., 2020), we have to consider the potential for current spreading to nearby sites and complex structures of the inner ear. Therefore, further research is needed to simulate calculations on current spread by HF-nVS to the vestibula and other areas, such as the cerebellum, and to directly record changes in the VOR function related to HF-nVS by detailed physiological experiments.

In standard VOR assessments, the clinician turns the patient’s head abruptly and unpredictably, roughly 15° in about 100 ms, and observes the compensatory eye movement response (Halmagyi et al., 2017). In another recent VOR assessment, Ischebeck et al. (2017, 2018) noted that the chair rotated for 33 s with an amplitude of 5.0° and a frequency of 0.16 Hz. This yielded five sinusoidal rotations of the chair with a peak velocity of 5.03°/s (Ischebeck et al., 2017, 2018). In our study, the participant was asked to hold their visual line to the fixation target and horizontally rotate their head as far as possible with a beep rhythm of 1 Hz. These frequencies and ranges of head rotation were faster and further than those used in previous studies (Halmagyi et al., 2017; Ischebeck et al., 2017, 2018). As a result of the settings of this experiment, the mean (SD) peak-to-peak angles of eye and head oscillations were slightly larger than other studies, at around 40–50° (0.8–1.0°). Although we cannot explain the mechanism by which the visual line deviation was not different between active and sham HF-nVSs, one possibility is that a large variation in peak-to-peak movements for eye and head (from faster and bigger movements) might obscure the difference. Additionally, previous studies recorded inter-ocular asymmetry torsion movements (Severac Cauquil et al., 2003). Given that we recorded horizontal eye movement only, we were unable to detect this. Therefore, further research is needed to record more precisely not only horizontal but also torsion eye movements in response to head movement.

A potential limitation of our study is the sample size, which was estimated using Hulley’s matrix method (Hulley et al., 1988). This method does not consider factors such as differences in age, sex, and baseline eye–head coordination performance and VOR function. Thus, a larger sample size is needed in further studies. These findings would be more widely representative with the addition of a detailed examination classifying participants by the above factors and the inclusion of a larger number of participants with normal VOR and VOR hypofunction.

## Future Perspective

Compensatory eye movements in response to head motion ensure the stability of the gaze and clear vision during motion, which is necessary to perform the activities of daily living, including playing sports and other tasks. Our findings highlight the potential of HF-nVS as a form of VOR training for patients with vestibular hypofunction. Although we investigated whether eye–head coordination was affected by HF-nVS, future studies should assess eye–head coordination in patients with brain injuries and investigate changes in VOR function using HF-nVS.

## Conclusion

In conclusion, our results show that by application of active HF-nVS, the time lag of eye and head movements was decreased compared by sham HF-nVS. Our results bring to light new ways of manipulating eye–head coordination with HF-nVS.

## Data Availability Statement

The original contributions presented in the study are included in the article/supplementary material. Further inquiries can be directed to the corresponding authors.

## Ethics Statement

The studies involving human participants were reviewed and approved by the Research Ethics Committee of the Tokyo Kasei University. The patients/participants provided their written informed consent to participate in this study.

## Author Contributions

All persons who meet authorship criteria are listed as authors, and all authors certify that they have participated sufficiently in the work to take public responsibility for the content. YM and MS contributed to conception and design of study, analysis and/or interpretation of data, and drafting the manuscript. YM, MS, NI, TO, KC, and Y-JW contributed to acquisition of data.

## Conflict of Interest

The authors declare that the research was conducted in the absence of any commercial or financial relationships that could be construed as a potential conflict of interest.
